# Changes in lumbar muscle diffusion tensor indices with age

**DOI:** 10.1093/bjro/tzae002

**Published:** 2024-01-13

**Authors:** Andrew D Weedall, Alexander Dallaway, John Hattersley, Michael Diokno, Charles E Hutchinson, Adrian J Wilson, Sarah C Wayte

**Affiliations:** Radiology Physics, Department of Clinical Physics and Bioengineering, University Hospitals Coventry and Warwickshire NHS Trust, Coventry, CV2 2DX, United Kingdom; Centre for Physical Activity, Sport and Exercise Sciences, Coventry University, Coventry, CV1 5FB, United Kingdom; Present Address: Faculty of Education, Health and Wellbeing, School of Health and Society, University of Wolverhampton, Wolverhampton, WV1 1LY, United Kingdom; Human Metabolic Research Unit, Department of Research and Development, University Hospitals Coventry and Warwickshire NHS Trust, Coventry, CV2 2DX, United Kingdom; School of Engineering, University of Warwick, Coventry, CV4 7AL, United Kingdom; Radiology Department, University Hospitals Coventry and Warwickshire NHS Trust, Coventry, CV2 2DX, United Kingdom; Radiology Department, University Hospitals Coventry and Warwickshire NHS Trust, Coventry, CV2 2DX, United Kingdom; Warwick Medical School, University of Warwick, Coventry, CV4 7AL, United Kingdom; Human Metabolic Research Unit, Department of Research and Development, University Hospitals Coventry and Warwickshire NHS Trust, Coventry, CV2 2DX, United Kingdom; Department of Physics, University of Warwick, Coventry, CV4 7AL, United Kingdom; Radiology Physics, Department of Clinical Physics and Bioengineering, University Hospitals Coventry and Warwickshire NHS Trust, Coventry, CV2 2DX, United Kingdom

**Keywords:** DTI, fat fraction, muscle, ageing

## Abstract

**Objective:**

To investigate differences in diffusion tensor imaging (DTI) parameters and proton density fat fraction (PDFF) in the spinal muscles of younger and older adult males.

**Methods:**

Twelve younger (19-30 years) and 12 older (61-81years) healthy, physically active male participants underwent T1_W_, T2_W_, Dixon and DTI of the lumbar spine. The eigenvalues (*λ*_1_, *λ*_2_, and *λ*_3_), fractional anisotropy (FA), and mean diffusivity (MD) from the DTI together with the PDFF were determined in the multifidus, medial and lateral erector spinae (ESmed, ESlat), and quadratus lumborum (QL) muscles. A two-way ANOVA was used to investigate differences with age and muscle and *t*-tests for differences in individual muscles with age.

**Results:**

The ANOVA gave significant differences with age for all DTI parameters and the PDFF (*P* < .01) and with muscle (*P* < .01) for all DTI parameters except for *λ*_1_ and for the PDFF. The mean of the eigenvalues and MD were lower and the FA higher in the older age group with differences reaching statistical significance for all DTI measures for ESlat and QL (*P* < .01) but only in ESmed for *λ*_3_ and MD (*P* < .05).

**Conclusions:**

Differences in DTI parameters of muscle with age result from changes in both in the intra- and extra-cellular space and cannot be uniquely explained in terms of fibre length and diameter.

**Advances in knowledge:**

Previous studies looking at age have used small groups with uneven age spacing. Our study uses two well defined and separated age groups.

## Introduction

The age-related degeneration of muscle is characterized by a loss of muscle mass and strength.^1^^,^^2^ One impact of these changes, collectively termed sarcopenia,^1^ is that it is associated with an increased risk of falls.^1^^,^^3^^,^^4^ The lumbar spinal muscles play an important role in rotation, flexion, extension, and stabilizing the spine^5^^,^^6^ and thus in maintaining balance. In sarcopenia, the loss of muscle mass is affected by factors other than age, including: the frequency and type of exercise;^7^^,^^8^ nutrition;^9^^,^^10^ and related co-morbidities (including diabetes mellitus, dementia, and cardiovascular disease^11^). The loss of muscle strength in sarcopenia is much greater than predicted by loss of muscle mass^1^ suggesting there may also be changes in the number, type, diameter, and organization of fibres within the muscle.^12–15^ The impact muscle ageing has on the quality of life for individuals in an ageing population means non-invasive methods are required to investigate the changes in muscle with age.

An increase in the fat content of muscle is a key component of the definition of muscle ageing^1^^,^^2^ and MRI determined proton density fat fraction (PDFF) is a quantitative measure of tissue adiposity. This measure, calculated from data acquired using chemical shift encoded water-fat MRI (Dixon imaging), indicates the global adiposity in an area of interest. Previous work has shown that muscle adiposity inversely correlates with strength^16^^,^^17^ and that this measure is a better predictor of muscle strength than cross-sectional area.^18^ Changes in muscle with age occur in conjunction with an increase in the mass of fat^12^^,^^16^ and connective tissue^19^ in the extra-cellular space, which contributes to a change in body composition. Whilst quantifying changes in the fat content of muscle is important in describing changes in muscle with age, it gives no information about changes in the muscle fibres or their organization.

Diffusion tensor imaging (DTI) is a quantitative method of assessing the movement of water molecules in tissue due to Brownian motion.^20^ The parameters used to quantify DTI are the eigenvalues (*λ*_1_, *λ*_2_, and *λ*_3_), from which two further parameters, fractional anisotropy (FA) and mean diffusivity (MD), are derived. The numerical values of these are determined by tissue microstructure constraining movement of water molecules. Age-related degeneration of muscle tissue has been shown to include changes in the structure of skeletal muscle,^15^^,^^21^^,^^22^ including fibre types and diameters.^12^ Previous work on the large muscles of the legs have attributed changes in numerical parameters from DTI to changes in muscle fibre type, size, and orientation.^14^^,^^23–25^ DTI has also been used to investigate how the tissue microstructure responds to exercise^26^ and injury.^27^ DTI parameters in the spinal musculature have been shown to be consistent with those from other muscles, poorly correlated with body mass index (BMI)^6^ and to predict muscle strength.^16^ Changes in the DTI parameters of the spinal musculature have also been shown to be associated with lumbar spinal disease.^5^

The study reported in this article aimed to investigate whether there were differences in the PDFF and DTI parameters of the spinal muscles between younger and older physically active adult males. Our hypothesis was that differences in the DTI parameters in the older group when compared to the younger group of participants would give evidence for DTI providing a non-invasive probe of changes in tissue microstructure reported in histological studies.^12^

## Materials and methods

This study was part of a wider investigation into muscle ageing in healthy male participants,^28^ which had ethical approval from the Coventry University Ethics Committee (reference P70399). The study was carried out in compliance with the Ethical Principles for Medical Research on Human Subjects set down in the Declaration of Helsinki by the World Medical Association. All participants provided written informed consent for the MRI data acquisition.

### Participants

Two groups of healthy, physically active adult male participants, one younger (*n* = 12) and one older (*n* = 12) were recruited as described by Dallaway et al.^28^ Inclusion criteria for this study were: healthy males aged 18-30 years; and healthy males aged 60 years or older. Exclusion criteria were: BMI outside of the range 18.5-29.9 kg.m^−2^; smokers; daily alcohol consumption; and an existing or past history of metabolic disease, neuromuscular disorders or musculoskeletal impairments that affect muscular strength. Table 1 shows the demographic data for the two groups.

**Table 1.** Participant demographic data giving the mean ± SD (range).

	Age group	
	Younger	Older
Age/years	24.7 ± 3.1 (19-30)	67.3 ± 6.0 (61-81)
Height/m	1.78 ± 0.1 (1.65-1.89)	1.74 ± 0.1 (1.63-1.87)
Weight/kg	76.4 ± 11.2 (57.4-91.9)	79.2 ± 10.8 (56.0-93.5)
BMI/kg m^−2^	24.1 ± 2.2 (21.1-27.1)	26.0 ± 2.7 (21.1-29.9)

### Data acquisition

Participants were scanned head-first, supine, on a GE Discovery 750 w 3 T scanner (GE Healthcare, Amersham, UK) using the 40-element spine array coil built into the scanning couch. Axial images were obtained from approximately the third lumbar vertebra to the fifth lumbar vertebra allowing visualization of the lumbar spinal muscles (quadratus lumborum [QL], erector spinae [lateral—ESlat and medial—ESmed] and multifidus [MF]) at these levels. T1_W_ imaging, T2_W_ imaging, Dixon imaging, and DTI were performed on each participant. Fat-only and water-only images were obtained of the lumbar spinal muscles using a 3D gradient echo IDEAL IQ sequence.^29^ The parameters used are shown in Table 2.

**Table 2.** IDEAL IQ imaging parameters.

Parameter	Value
TR/TE	16.6/5.1 ms
Receiver bandwidth	111 kHz
FoV	24 cm^2^
Slice thickness	4 mm
Matrix	224×224
Flip angle	6°
Number of averages	4
Acquisition time	8 min 12 s

The DTI was performed using a single-shot echo planar imaging technique with spectral fat saturation using the parameters shown in Table 3.

**Table 3.** DTI imaging parameters.

Parameter	Value
TR/TE	800/63 ms
Receiver bandwidth	250 kHz
FoV	24 cm^2^
Slice thickness	4 mm
Matrix	96×96
Diffusion directions	15
*b*-values	0, 450 s.mm^−2^
Number of averages	4
Acquisition time	8 min 40 s

The image sets were acquired sequentially without repositioning the subject with all scans performed between the same axial start and end points, with the same field of view and with the same slice thickness. In addition, a scanner utility “copy coverage” was used to ensure all images had the same inferior-superior coverage. Each slice in the 30 image sets were interpolated to 256×256 pixels before being stored in DICOM format. All image processing was done on these DICOM images.

### Data analysis

**Figure 1.**
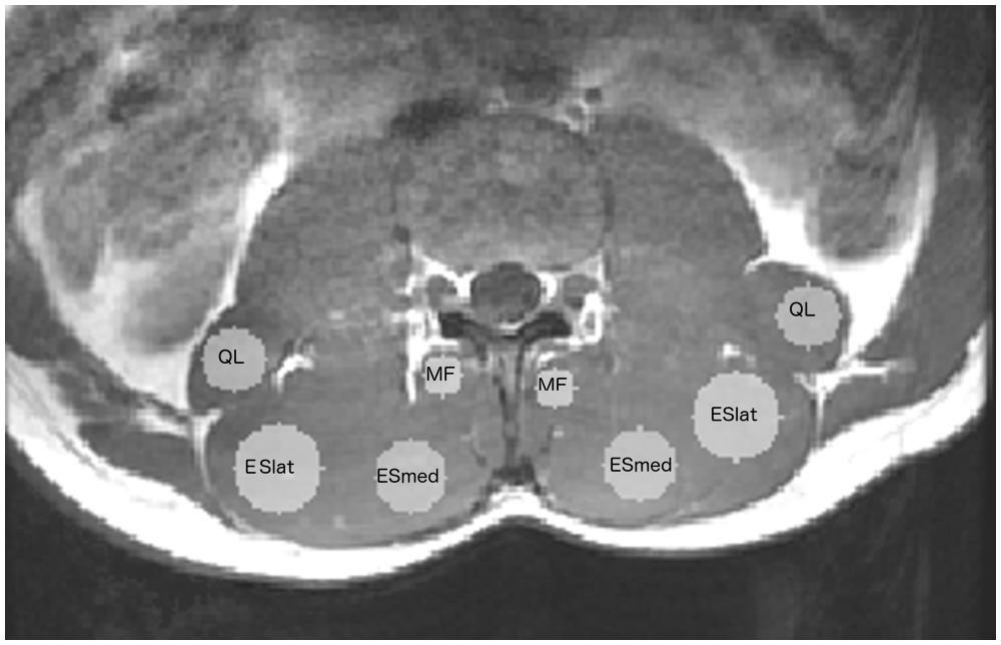
An example of the circular regions of interest (ROIs) in the muscles studied on the L3ep slice of a T1_w_ image. The diameter of the ROI for each muscle was the largest that could be fitted into all four sites (right and left for L3ep and L4ep) excluding regions of fat for that muscle.

For each participant in the study, the slice including the superior end plate of the third lumbar vertebra (denoted L3ep) and the slice including the superior end plate of the fourth lumbar vertebra (denoted L4ep) were identified manually (by A.J.W. and A.D.W.) from the T1_W_ and T2_W_ images using ImageJ (https://imagej.nih.gov/). All images were then converted from DICOM to NIfTI format using the Matlab routine dicm2nii (https://github.com/xiangruili/dicm2nii). Using FSLeyes (https://fsl.fmrib.ox.ac.uk/fsl/fslwiki/FSLeyes), circular regions of interest (ROIs) were drawn on slices in the T1_W_ image slices at L3ep and L4ep on the left and right side of the body in the muscle being studied. Binary masks were then created for these ROIs (Figure 1). For each participant the diameter of the ROIs was the same for all four sites of the same muscle and was the largest possible ROI contained wholly in the muscle avoiding obvious regions of fat.

### Proton density fat fraction

The PDFF data were determined using a method based on previous work^30^ in the ROIs created for each muscle site. If *f*_i_ and *w*_i_ are pixels from the fat only and water-only images, respectively, in an ROI then the fat fraction (PDFF) for that pixel, *ff*_i_ is given by:

(1) $${ff}_{i}\;=\;\frac{f_{i}}{f_{i}+w_{i}},$$

and the average PDFF for the ROI, PDFF_roi_, is given by:

(2) $${\mathrm{PDFF}}_{\mathrm{roi}}\;=\;\frac{\sum_{i=1}^{N_{i}}{ff}_{i}}{N_{i}}.$$

The PDFF for each muscle studied is taken as the average value of PDFF_roi_ for all ROIs in that muscle.

### Diffusion tensor imaging

The eigenvalues of the diffusion tensors were determined within the circular ROIs using the tensor fitting model within DIPY (https://dipy.org) from which the FA and MD were calculated. The mean values for these parameters were then calculated for each ROI. From these, average values for the eigenvalues, FA, and MD were then calculated for each muscle for each participant.

### Statistical analysis

Statistical comparisons of *λ*_1_, *λ*_2_, *λ*_3_, FA, MD, and PDFF between age groups and muscles were performed in R (https://www.r-project.org) using a two-way ANOVA. Where there was a statistically significant difference between muscles a Tukey HSD follow-up analysis was performed between pairs of muscle sites. In addition, differences with age were further investigated to determine whether these were muscle dependant using *t*-tests to compare the age groups for each muscle independently. Statistical significance was taken as *P* < .05 throughout. Corrected probability values are reported for the Tukey HSD analysis and a Bonferroni correction for multiple comparisons was applied to probability values from the *t*-test analyses.

## Results

For the ESlat, ESmed, and MF muscles, ROIs could be created for all four sites, however, for the QL muscle it was not possible to create ROIs at the L4ep level in all participants. In four participants from the younger group and all participants in the older age group, the L4ep was below the superior surface of the iliac fossa and hence below the inferior extent of the QL muscle. Thus, no measurement for the QL muscle at the L4ep level could not be made and DTI parameters for the QL are only from the L3ep level.

The diameter of the ROIs was consistently higher in the younger group than the older group (Table 4), primarily due to a greater volume of fat within the muscle of the older group. With the exception of the ROI for ESmed, the variation in the diameter of the ROIs between subjects was greater in the older group than in the younger group.

**Table 4.** The mean ± SD for the diameters of the ROIs in pixels (0.96 mm) for the different muscles.

Muscle	Younger	Older
Multifidus (MF)	3.33 ± 0.65	1.92 ± 0.79
Erectus spinae—lateral (ESlat)	11.25 ± 1.22	7.67 ± 1.87
Erectus spinae—medial (ESmed)	9.50 ± 1.83	7.58 ± 1.24
Quadratus lumborum (QL)	14.25 ± 0.87	12.50 ± 1.73

Abbreviations: SD = standard deviation; ROI=region of interest.

**Figure 2.**
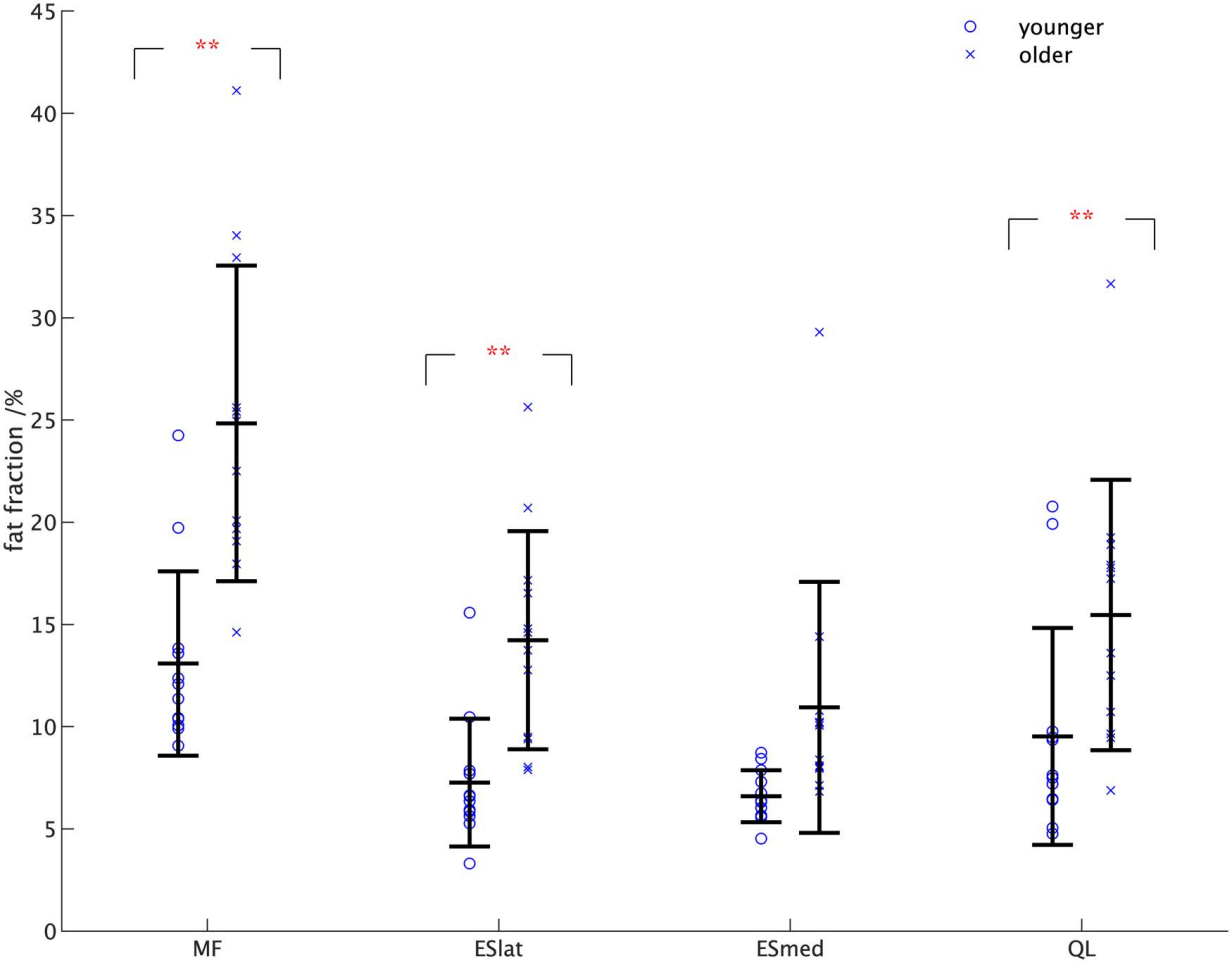
The individual values for the PDFF within the ROIs. The values of the mean±standard deviation for each muscle (Table 5) are shown together with the results of the *t*-test comparisons between muscles (*P < .05, **P < .01). Abbreviations: PDFF = proton density fat fraction; ROIs = regions of interest.

Figure 2 shows the individual PDFF values for each of the muscles studied together with the mean and SD from Table 5.

**Table 5.** The mean ± SD of the PDFF for each muscle expressed as a % in each age group.

	MF	ESlat	ESmed	QL
Younger	13.09 ± 4.51	7.27 ± 3.13	7.23 ± 1.36	9.52 ± 5.31
Older	24.83 ± 7.72	14.23 ± 5.34	9.69 ± 2.09	15.46 ± 6.61

Abbreviations: SD = standard deviation; PDFF = proton density fat fraction.

The two-way ANOVA on these data showed a statistically significant difference in PDFF with both age (*P* < .01) and muscle (*P* < .01), with a significant interaction between age and muscle (*P* < .05). The only significant differences between pairs of muscles in the follow-up analysis to the ANOVA were between ESmed and MF (*P* < .01), ESlat and MF (*P* < .01), QL and MF (*P* < .01), and between ESmed and QL (*P* < .05) (Table 6).

**Table 6.** Post-ANOVA analysis of differences in PDFF between pairs of muscles (-*P* > .05, *P < .05, **P < .01).

Muscle 1	Muscle 2	*P*
MF	ESlat	**
MF	ESmed	**
MF	QL	**
ESlat	ESmed	−
ESlat	QL	-
ESmed	QL	*

Abbreviations: ANOVA = analysis of variance; PDFF = proton density fat fraction.

From Figure 2, it can be seen that the mean PDFF was higher in the older group with *t*-tests giving statistically significant differences for MF, ESlat, and QL at *P* < .01 but not for ESmed (*P* > .05).

**Figure 3.**
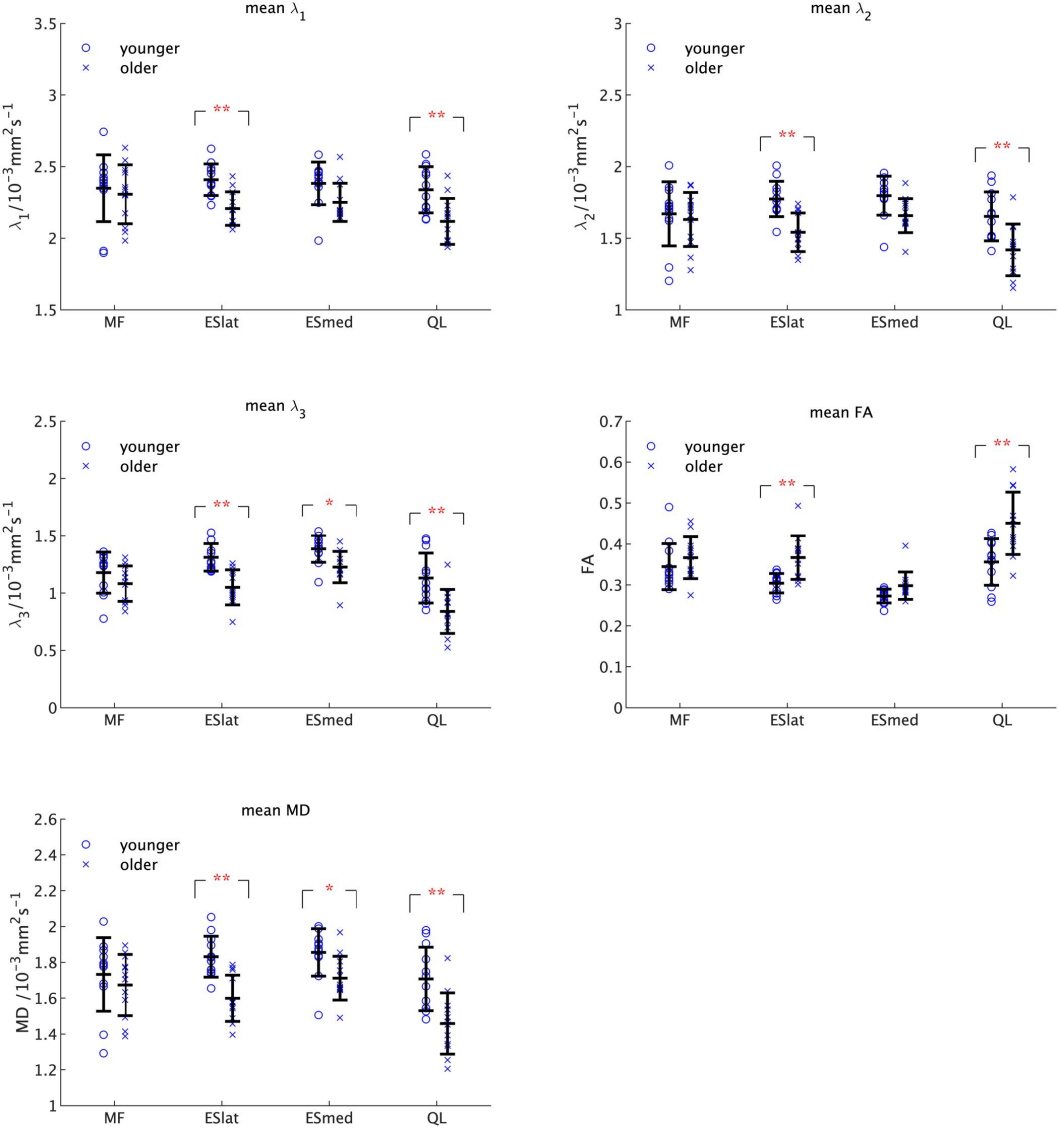
The individual values of the DTI parameters for each muscle. These are shown together with the mean and standard deviations for each muscle in each age group (Table 7). Results of the *t*-test analysis of differences between age groups for each muscle are shown (*P < .05, **P < .01). Abbreviation: DTI = diffusion tensor imaging.

Figure 3 shows individual values for each of the DTI parameters with the mean ± SD values from Table 7 superimposed on them.

**Table 7.** The mean ± SD of the DTI parameters for the younger and older age groups.

Muscle position	Age group	DTI parameter				
		*λ*_1_	*λ*_2_	*λ*_3_	FA	MD
		/10^−3^ mm^2^ s^−1^	/10^−3^ mm^2^ s^−1^	/10^−3^ mm^2^ s^−1^		/10^−3^ mm^2^ s^−1^
Multifidus	Younger	2.35 ± 0.23	1.67 ± 0.22	1.18 ± 0.18	0.34 ± 0.06	1.73 ± 0.21
(MF)	Older	2.31 ± 0.21	1.63 ± 0.19	1.08 ± 0.15	0.37 ± 0.05	1.67 ± 0.17
Erectus spinae—lateral	Younger	2.41 ± 0.11	1.77 ± 0.12	1.31 ± 0.12	0.30 ± 0.02	1.83 ± 0.11
(ESlat)	Older	2.21 ± 0.12	1.54 ± 0.13	1.05 ± 0.15	0.37 ± 0.05	1.60 ± 0.13
Erectus spinae—medial	Younger	2.38 ± 0.15	1.80 ± 0.14	1.39 ± 0.12	0.27 ± 0.02	1.86 ± 0.13
(ESmed)	Older	2.25 ± 0.13	1.66 ± 0.12	1.23 ± 0.14	0.30 ± 0.03	1.71 ± 0.12
Quadratus lumborum	Younger	2.34 ± 0.16	1.65 ± 0.17	1.13 ± 0.22	0.36 ± 0.06	1.71 ± 0.18
(QL)	Older	2.12 ± 0.16	1.42 ± 0.18	0.84 ± 0.19	0.45 ± 0.08	1.46 ± 0.17

Abbreviations: SD = standartd deviation; DTI = diffusion tensor imaging.

The results of the 2-way ANOVA on these data gave a statistically significant difference for all the DTI parameters with both age (*P* < .01) and between muscles (*P* < .01) for all measures except *λ*_1_. There was no interaction between age and muscle for any of the DTI parameters (*P* > .05).

The results for the post-ANOVA analysis of differences in the DTI parameters between pairs of muscles are summarized in Table 8. It is interesting to note that the number of significant differences between muscles is much larger for *λ*_3_ than for the other eigenvalues.

**Table 8.** Post-ANOVA analysis of differences between pairs of muscles (-*P* > .05, *P < .05, **P < .01).

Muscle 1	Muscle 2	DTI parameter				
		*λ*_1_	*λ*_2_	*λ*_3_	FA	MD
MF	ESlat	–	–	–	–	–
MF	ESmed	–	–	**	**	–
MF	QL	–	–	**	**	**
ESlat	ESmed	–	–	**	**	–
ESlat	QL	–	–	**	**	**
ESmed	QL	–	*	**	**	**

Abbreviation: ANOVA = analysis of variance.

The *t*-tests between pairs of muscles (Figure 3) showed statistically significantly lower values for all three eigenvalues in the older group for ESlat and QL (all *P* < .01). ESmed only gave a significant difference for *λ*_3_ (*P* < .05). There were statistically significantly higher values of FA in the older group for ESlat and QL (both *P* < .01) but not for ESmed (*P* > .05) and statistically significantly lower values of MD in the older age group for ESlat and QL (*P* < .01), ESmed (*P* < .05) but not for MF.

## Discussion

As expected, the results show an increase in PDFF in the older group in all muscles, consistent with descriptions of muscle ageing^1–3^ and with studies finding a negative correlation between muscle mass and age,^22^ including in the muscles, we studied.^31^ Whilst the PDFF was higher in the older group for all muscles studied, it was not statistically significant in all muscles. This together with the significant interaction between age and muscle from the ANOVA analysis suggests that changes in PDFF in the spinal musculature with age are muscle dependant.

For the DTI parameters, our results showed lower values for all of the eigenvalues and MD in the older group for all muscles studied, with the differences being statistically significant for all these measures in ESlat and QL but only for *λ*_3_ and MD in ESmed. The difference for MF did not reach statistical significance for any of these measures. The FA was shown to be higher in the older group for all muscles studied, again with the differences being statistically significant in all the muscles studied except for ESmed and MF. Our results for the eigenvalues were consistent with the findings of one previous study,^14^ but not another where increases in all eigenvalues with age were reported.^13^ Consistent with our findings, not all differences with age reached statistical significance in all muscles included in these studies.^13^^,^^14^ Another study only reporting FA and MD found no significant change in FA with age^32^ inconsistent with our findings and one other study,^13^ but not with the findings from another study.^14^ However, the value of MD from the same study^32^ showed a significant increase with age, which is inconsistent with our findings. Loss of muscle strength is associated with muscle ageing^1^^,^^12^ and a positive correlation has been reported between the eigenvalues, MD and radial diffusivity (RD = [*λ*_2_ + *λ*_3_]/2), and strength in the spinal musculature.^16^ Therefore, a decrease in the eigenvalues with age would be expected, consistent with our results. Theoretical work concluded that increasing fibre diameter is the largest determinant of increasing values of *λ*_2_ and *λ*_3_,^25^ a conclusion supported by a negative correlation between the proportion of type I fibres and RD reported in an experimental study^33^ as type II fibres have a larger diameter than type I fibres. This together with histology studies showing a preferential reduction in the diameter of type II fibres with age^12^^,^^34^ further supports our finding of reduced values of *λ*_2_ and *λ*_3_ in the older group. The lack of consistency in findings with age between studies and even between muscles within the same subject^13^^,^^14^ needs an explanation. Whilst understanding the results obtained for PDFF in terms of the expected changes with ageing is straightforward, the same is not true for the DTI parameters. Although the number of studies comparing DTI parameters with age is small, there is a larger body of literature on theoretical models,^25^ and studies aimed at correlating DTI measures with muscle strength^16^ that contribute to the interpretation.

Ageing reduces the number of fibres^15^ and shows selective atrophy of the larger diameter, type II, fibres,^35–37^ reducing the average diameter of the fibres, increases the volume of the extra-cellular space, which must have an impact on the eigenvalues. Sinha et al,^13^ based on the work of Galban et al^38^ and Krampinos et al,^39^ proposed a weighted sum model based on “fibre fraction” to describe how the fibres and extra-cellular space contribute to a final value for *λ*_1_, *λ*_2_, and *λ*_3_. This model is based on the view that the eigenvalues calculated from images are the result of the properties of the fibres and the extra-cellular compartment. An assumption implicit in the weighted sum model^13^ is that molecular movement in the extra-cellular space is isotropic, which leads to the logical conclusion that *λ*_1_ should remain unchanged or increase with ageing as the fat content of muscle increases.^1^ However, the extra-cellular space is not homogeneous but contains fat cells, connective tissue, and necrotized fibres—a content that will increase with age^12^^,^^40^ giving barriers that restrict movement of water molecules hence reducing all 3 eigenvalues. Thus, this linear representation of the fibres and extra-cellular space using a single value, the (cross-sectional) fibre fraction, to describe the properties of both the fibres and the extra-cellular space appears to be an over-simplification. However, this model does establish the important principle that changes in the value of each eigenvalue with age (and consequently FA and MD) are the result of changes in both the fibres and the extra-cellular space.

## Conclusion

We have shown an increase in PDFF and a decrease in eigenvalues in the lumbar muscles of older individuals. The components of muscle ageing, which include changes in both the fibres and the extra-cellular space occur at different rates in different individuals over a long period of time. Therefore, it is perhaps an over-simplification to explain the changes in eigenvalues in terms of fibre diameter and length. Moreover, current knowledge means it is impossible to uniquely relate changes in eigenvalues to changes in fibres and the extra-cellular space.

## Data Availability

The data underlying this article will be shared on reasonable request to Sonia Kandola, Research Governance and Quality Manager. Research and Development Department, University Hospitals Coventry and Warwickshire NHS Trust, Coventry, CV2 2DX, UK.
